# Case reports of new-onset eating disorders in older adult cancer survivors

**DOI:** 10.1186/s40337-021-00522-5

**Published:** 2021-12-24

**Authors:** Dori Rakusin, Kate O’Brien, Michael Murphy

**Affiliations:** 1 Psychiatry Department, The Prince of Wales Hospital, Randwick, NSW 2031 Australia; 2 Nutrition and Dietetics Services, The Prince of Wales Hospital, Randwick, NSW 2031 Australia; 3 School of Psychiatry, UNSW, Randwick, NSW 2031 Australia

**Keywords:** Disordered eating behaviours, Eating disorder, Case report, Cancer surgery, Weight loss, Gastrointestinal

## Abstract

**Background:**

It is unusual for a de novo eating disorder to emerge in late adulthood. Across a number of years, a number of patients were identified who experienced severe and unexpected weight loss post curative management of an upper abdominal cancer (i.e., cancer survivors). Each of the patients was readmitted to the tertiary surgical hospital due to complications of severe malnutrition. Each presentation was initially considered to have a major physical health problem (such as cancer recurrence). Each required extensive investigations and multidisciplinary team involvement and were later conceptualised as a new-onset (in later adulthood) eating disorder that emerged post curative cancer treatment. The team wished to better understand this group and/or characterise and/or inform the scientific community of this phenomena if not already well described.

**Literature review:**

The review identified that the re-emergence of pre-existing eating disorders at the time of cancer treatment was described; however, there was no identification of similar new-onset in later adulthood cases of eating disorders in cancer survivors in the medical literature.

**Review of the cases:**

Once ethics and consent was obtained, then the clinical course of four complex individuals were reviewed, including the use of a multidisciplinary Delphi review process, to understand commonalities and then plot a common care pathway with potential intervention points.

**Case presentations:**

Common factors identified among the four patients included the lack of a physical health (organic) cause to the weight loss and ongoing weight loss despite intense multidisciplinary care. All had abnormal attitudes and behaviours relating to food, nutritional rehabilitation and/or recovery. None returned to a healthy weight and/or healthy eating despite extensive team input. The presentations were ultimately conceptualised as having severe disordered eating behaviours and in at least three cases met criteria for a formal eating disorder. The cohort had similar psychosocial characteristics including low socioeconomic status and complex family dynamics. None had prior formal psychiatric care. The outcomes were poor; one patient died, another required admission to a specialist eating disorder admission with a subsequent relapsing remitting course, and the remaining two had complicated chronic courses.

**Conclusion:**

Similar cases may be underreported. If identified earlier, there may be a role for intervention to prevent high morbidity and mortality and to support clinical teams managing similar complex patients.

## Background

Feeding and eating disorders (FEDs) have at their core entrenched disordered eating behaviours (DEBs). Diagnostic and Statistical Manual of Mental Disorders (DSM)-5 outlines criteria for six specific FEDs, namely anorexia nervosa (AN), Bulimia Nervosa, Binge-Eating Disorder, Avoidant/Restrictive Food Intake Disorder (ARFID), Pica and Rumination Disorder [1]. Whilst most FEDs have either a childhood or adolescent/young adult onset, there are case reports of late-onset FEDs (generally understood as onset after age 40) [2]. This case series identifies several patients who developed FEDs post-surgery. We wish to better conceptualise their presentations.

## Rational for this study

The authors identified a group of older adult patients who were readmitted to a surgical ward in a general hospital due to complications of severe malnutrition. The patients were unable to maintain their expected weight and the malnutrition occurred many months, and in one case years, post treatment for cancer (all had major surgery and/or chemo-radiotherapy for the treatments of upper gastrointestinal cancers). The weight loss was not attributable to cancer recurrence, surgical complications, anatomical anomalies, or other physiological explanations. Over time the patients required intensive multi-disciplinary support (including nursing, dietitian, surgical teams, eating disorder clinicians etc.) with subsequent consultation to psychiatry for opinion and management advice, given their complexities. *Firstly,* we wished to clarify whether such a cohort was previously identified in the scientific literature. *Secondly*, we wished to investigate whether commonalities to their presentation and/or clinical characteristics existed. *Lastly*, we supposed that similar presentations occur in other hospitals and so wished to raise awareness.

## Methods

Firstly, a literature review was completed in late 2019 looking to identify reports that had all three of the following characteristics:

- New (i.e., de novo) onset in later adulthood (> 40 years old),
- Severe (i.e., requiring hospitalization) feeding and eating disorders (FEDs) of psychiatric origin,
- In cancer survivors post-surgery.

Secondly, a case-based review of the identified suitable patient charts was undertaken with completion of a summary of common characteristics/ factors to better characterise the cohort.

Lastly, several rounds of consultation between the authors, utilising a Delphi method [3], occurred to generate the common care pathway. The findings are presented in an accessible format, up to and including possible points of intervention for the multi-disciplinary team.

## Results

### Literature review

The literature review did not identify similar case reports meeting all three of the criteria of (a), (b) and (c) as outlined above in methods.

There is however scientific literature showing case series of “Synopsis of reported cases of anorexia nervosa associated to cancer” in children and young people [4]. There are articles outlining the multifactorial, physiologically manifested, nature of anorexia in cancer [5]. Similarly, there is further information, including systematic reviews, of the physiological causes of malnutrition post curative surgery for cancer (e.g., gastrectomy and/or oesophagectomy) [6]; hence these causes are driven through physiological or organic means, rather than having a psychiatric origin.

Similarly single (i.e., non-reproduced) articles in this area discuss how common body image disorders are in the cancer setting [7]. Other papers identify psychological components, such as fear, anxiety, disappointment and depression which may contribute to poor postoperative nutritional intake in cancer patients [8]; but these papers do not mention a psychiatric syndrome, rather they explain the range of emotional responses that may occur.

Lastly, there are case series of later adult onset FEDs post-surgery; however not in cancer survivors. Within the late-onset subset of patients with eating disorders, case reports emerged in the literature over 20 years ago of a possible new-onset, or de novo*,* FED post bariatric surgery (commonly known as weight loss surgery), with some authorship groups proposing the need to formally recognise a new FED for patients who develop an AN-like illness post bariatric surgery, *Post-Surgical Eating Avoidance Disorder* [9].

Hence, overall there was no identified scientific literature identifying all three key aspects of the cases i.e., (a) new-onset in late adult hood, (b) eating disorder (severe malnutrition mediated through psychiatric and/or psychological means and severe enough to warrant hospitalization) and, (c) occurring in cancer survivors.

### Decision to detail the case series

Due to the lack of similar case reports in the scientific community, it was deemed appropriate to consider reporting the cases. Appropriate correspondence with the hospital ethics board occurred. For the purpose of this case series, five patients were identified over the course of two years (2018 and 2019). When approached retrospectively only four consented for the study. Written consents were obtained (from three patients and in the fourth case from the deceased’s next-of-kin).

### Case presentations

#### Case 1

A sixty-seven year-old, unemployed female living with her ex-partner was diagnosed with early stage oesophageal cancer upon investigation for dysphagia. She underwent preoperative combined chemo-radiotherapy to shrink the tumour and subsequently had upper abdominal surgery (Ivor-Lewis oesophagectomy). She had early post-surgical weight loss and was discharged from hospital with nutrition via a feeding tube. On community dietician follow-up she admitted to non-adherence to the feed schedule and reported stress and anxiety associated with feeding. She became upset when the option of increasing feed rates was raised. She had repeat presentations to the emergency department with dislodged feeding tube. Due to concerns about a possible physical problem (stricture) impacting on her oral intake, a preventative dilatation was performed. Despite this, she continued to lose weight in the community, became increasingly fussy about food textures and flavours and justified her minimal intake to family and dieticians. Eight months post initial surgery she was admitted with severe bradycardia and hypotension secondary to malnutrition. A nasogastric tube was then inserted for the purpose of refeeding.

#### Case 1 - Mental health assessment

She was initially referred to the consultation liaison (CL) psychiatry team due to concerns around stress/anxiety. She was initially diagnosed with major depressive disorder with anxious features. Though her difficulties swallowing had some structural basis, they were exacerbated by anticipatory anxiety about choking. Conflict at home over her oral intake led to heated arguments, increased stress and subsequent loss of appetite. Aside from pre-existing moderate obsessive tendencies, she had no known psychiatric or substance use history, nor pertinent family history. Subsequent reviews revealed full remission of the major depressive features and acknowledgement of typical anorexic cognitions including a fear of weight gain. In time, she was noted to tamper with nutrition given via tube feeding and over-exercise. Later, a formal diagnosis of Anorexia Nervosa was made. Over the next two years she required multiple admissions for complications of malnutrition including ultimately an admission to a Specialist Eating Disorder Unit. Further medical admissions have occurred due to medical complications of severe malnutrition.

#### Case 2

A sixty-nine year-old retired woman, living with her husband and acting as his career as he had a dementia illness, was diagnosed with early stage oesophageal cancer. She underwent preoperative chemotherapy, followed by upper abdominal surgery (gastrectomy, lower oesophagectomy and roux-en-y). She had a complicated post-operative course with multiple admissions to intensive care owing to wound infection. She was commenced on total parenteral nutrition (TPN, nutrition directly into the vein), which was later transferred to nutrition via a feeding tube. She was discharged four months post-operatively, not yet meeting her nutritional needs orally. On community dietician follow-up, she admitted to non-adherence to feeds and reported simply being unable to eat on most days. She had multiple presentations to the emergency department (ED), complaining of uncontrollable dry-retching, preventing her from complying with feeds via the feeding tube. The dry-retching was not observed during any ED assessments but further weight loss was noted. Eight months post-surgery, she warranted readmission for nutritional rehabilitation.

#### Case 2 - Mental health assessment

She was initially referred to CL psychiatry on account of the disparity between her stated intentions to recover and her refusal to comply with advice regarding feeding. No formal psychiatric diagnosis was given at the time, nor suggestion made about personality vulnerabilities. She had no previous formal psychiatric diagnoses, bar a solitary depressive episode in her twenties, nor history of substance use. She attributed her inability to eat as being secondary to a reported alteration in taste (dysgeusia), early satiety and anorexia. Over time she had an increasingly narrow repertoire of foods she would agree to eat, became increasingly non-compliant with nursing and dietician advice and refused all nutritional supplements. More than a year post surgery, she was given a diagnosis of an Atypical Anorexia Nervosa. Despite attempts at ongoing care, she remains severely underweight and has now disengaged with all follow-up.

#### Case 3

A sixty-two year-old married man, living with his ageing mother and second wife was diagnosed with oesophageal cancer. He underwent an upper abdominal surgery (Ivor Lewis oesophagectomy). Though he had expected post-surgical weight loss, he ultimately made a good recovery. Nine years later however, he presented with complications of severe, rapid weight loss attributed to nausea. After the possibility of cancer recurrence was excluded, he was discharged with advice to take nutritional supplements, use anti-emetics and follow-up with a community-based dietician. Over the following six weeks, he had multiple presentations to ED with various unrelated somatic complaints. Further weight loss was noted on each occasion. He was eventually re-admitted for delivery of nutrition via a feeding tube and re-establishment of normal oral intake. Again, physical health problems such as motility gut problems and strictures were excluded.

#### Case 3 - Mental health assessment

He was referred to psychiatric services for assessment of atypical eating behaviours and related cognitions; extreme anxiety around consumption, narrowing of food repertoire, avoidance of particular food groups and cutting food into minute pieces. Though he had no formal psychiatric history, there was evidence to suggest he had developed features of an Illness Anxiety Disorder after his cancer surgery many years prior. He had no drug and alcohol use, nor history of the same. At the time of first psychiatric assessment, he was given a diagnosis of Somatic Symptom Disorder and was seen on a regular basis by a CL clinical psychologist, for cognitive behavioural therapy. He went on to total refusal of all oral intake and anti-emetics (despite his chronic complaint of nausea). There was marked cognitive dissonance between his DEB and an enduring wish to live. He appeared to understand the outcome of chronic food refusal (i.e., death) and repeatedly reported wanting to recover. He later had a revised diagnosis of Avoidant Restrictive Food Intake Disorder. He made little effort to engage with psychological/psychiatric services and made no improvement in his oral intake. He ultimately died from infection superimposed on extreme frailty.

#### Case 4

A 61-year-old partnered male, living alone, presented with a diagnoses of stomach cancer (gastric adenocarcinoma). He underwent upper abdominal surgery (radical gastrectomy). Post-operatively, he was unable to tolerate any oral intake and was discharged with a feeding tube in place. The feeds provided less than fifty percent of his nutritional needs. As an outpatient, he was non-compliant with the prescribed feed schedule and did not adhere to the recommended post-gastrectomy diet. He restricted his oral intake to sugar-packed carbonated beverages and did not take the pancreatic enzyme replacement (Creon®), nor the prescribed dietary supplements. At community-based dietician review, he was noted to have lost more weight. There were numerous incidences of unexplained feeding tube dislodgement. He had three emergency department presentations due to nausea, with further weight loss noted on each occasion. He was eventually admitted due to extreme frailty and uncontrollable nausea. Repeat investigations were all unremarkable.

#### Case 4 - Mental health assessment

Referral to CL psychiatry for concern around dietary non-compliance was made eight months post initial surgery. Though no formal diagnosis was made initially, he was noted to have an anxious preoccupation with food planning, to the extent that he developed total food avoidance.

There was an obvious discrepancy between his stated fears of further weight loss as a precursor to death and his wish to recover. His therapy-interfering behaviours persisted despite traumatic memories of his mother’s cachexia secondary to cancer resulting in her death. He demonstrated limited insight into the contradictory nature between his cognitions and behaviours thought to reflect very poor health literacy. He had a prolonged in-patient admission for nutritional rehabilitation which was unsuccessful. Later in that admission a diagnosis of an Atypical Anorexia Nervosa was made. Despite not recovering, he was discharged due to limited benefit of ongoing inpatient care. Clinical acumen deemed that he was unlikely to engage with any further service and had poor prognostic indicators.

### Results-common characteristics

Table 1 outlines the oncological interventions, nutritional, demographic and psychosocial characteristics of the four patients. Key common factors included all four having (excessive) weight loss 12 months postoperatively i.e., our case series range of weight loss at the one year post-operative mark (15.7–37.5% body weight loss) was generally higher than the *expected* body weight loss of 15–17% at postoperative 1-year [6] for an oesophagectomy and 10–20% after gastrectomy [10]. Premorbid, only Case 1 had lifelong low weight (that neither warranted formal intervention nor caused family concern), with the remaining three being normal to mildly overweight across their lifetime.

**Table 1:** Case overviews and common characteristics

Domain	Commonalities
Problem behaviours leading to re-admission due to complications of severe malnutrition. Diagnosis	*Case 1* 67yo woman, cancer survivor. She was non-adherent to community nutrition advice She was poorly adherence to parenteral nutrition plan and had a decreasing food repertoire There was a gradual, continued weight loss post discharge such that she was re-admitted 8 months post initial surgery Formal diagnosis of Anorexia Nervosa later made. Repeat admissions and presentations *Case 2* 69yo woman, cancer survivor. She warranted a prolonged admission for weight purposes post initial cancer surgery including TPN Thereafter, she was non-adherent to her community parenteral feeding plan and reported no desire to eat She suffered from subjective dry-retching and ongoing weight loss. She was readmitted 12 months post initial surgery Diagnosis of Atypical Anorexia Nervosa around that time. Unclear outcome due to disengagement *Case 3* 62yo man, cancer survivor. He had significant anxiety and somatic concerns post initial cancer treatment Many years later, he had severe, rapid weight loss due to subjective nausea and bodily complaints He was also then noted to have unusual and severely rigid eating patterns and behaviours Later diagnosis of Avoidant Restrictive Food Intake Disorder. He ultimately died of frailty *Case 4* 61yo man, cancer survivor. He was non-compliant post-operatively with both his parenteral feed schedule and diet plan Over the course of months he had limited insight into his continued weight loss, with likely death and near total food avoidance At the time of discharge, Atypical Anorexia Nervosa diagnosis was made with concerns for a poor prognosis
Nutritional history before referral to psychiatry	All had excessive weight loss postoperatively (above expected post-operative weight loss) Most were unable to maintain adequate nutrition at time of initial surgical discharge
Psychiatric factors (referral process, diagnosis, prognosis, outcome)	Perhaps understandably, all had a late referral to see psychiatry given complexity However, all patients were agreeable to psychiatric initial engagements Longitudinal (i.e., repeat) assessments and a multidisciplinary approach was needed for diagnosis None returned to either a healthy weight and/or healthy eating behaviours despite extensive team care
Psycho-social factors	Pre-morbid (pre-cancer) mild maladaptive coping issues were identified Complex family dynamics (family position of mediator or carer) and generally lower socioeconomic standing were noted
Key absent findings	None had a prior eating disorder diagnosis No other relevant psychiatric history/drug or alcohol concerns No concerns for clinical depression or psychotic illness at time of FED consideration No psychiatric medications at time of initial psychiatric referral
Cancer/physical health	Major upper abdominal surgery resulting in removal of all cancer Most had chemotherapy and/or radiotherapy treatments before surgery to shrink the tumour All had no physical health findings that could explain the weight loss—i.e., no return of cancer, no strictures etc

*FED* feeding and eating disorder, *TPN* total parenteral nutrition

There was some variation in the type of DEBs. All presented with a disparity between their stated wish to recover (i.e., to gain weight) and their observed actions of further restriction, resulting in malnutrition. In contrast to anorexia nervosa, there were no classic body image concerns expressed; however, like anorexia nervosa, each patient downplayed, or were unable to appreciate, the concerns of their clinicians and family. Some patients were in keeping with “classic” anorexia nervosa with therapy interfering behaviours such as tampering with nasogastric feeding; however, other patients were simply non-compliant with all basic dietary advice and refusal of all supplements despite obvious life-threatening malnutrition. There were confirmed cases of a compensatory behaviour (one over exercising on the ward and another patient had an atypical purge pattern). To our knowledge there was no laxative abuse.

From a psychiatric viewpoint, two had pre-existing formal psychiatric diagnoses (one case of major depressive illness, one case of alcohol use disorder, both were in remission at the time of consideration of the FED). There were no past or present psychotic or cognitive disorders. Early life adversity and premorbid personality vulnerabilities were identifiable in all four patients. None were taking psychotropic medications at time of initial psychiatric referral.

The outcome for the patients were one dying, one proceeding to an admission to the public specialist eating disorder unit, and then as per the remaining two, continuing to have ongoing malnutrition and intermittent general medical-surgical admissions with general disengagement from mental health/ dietitian/ support services.

### Results-common pathway

**Fig. 1:**
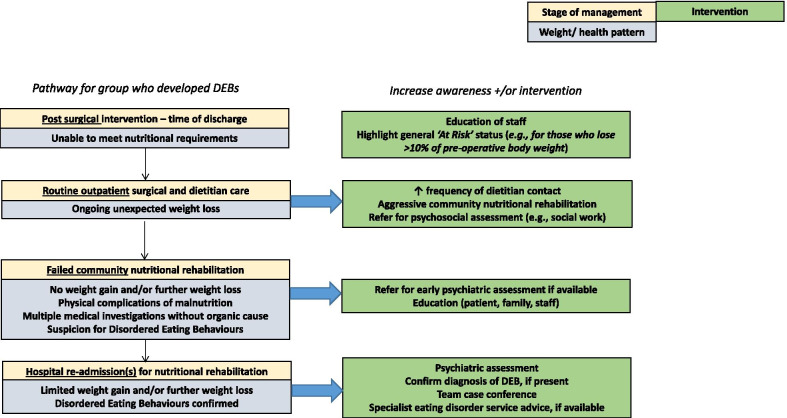
Common pathway and potential intervention points in patients with disordered eating behaviours (DEB) post-surgery for upper abdominal cancer

A pathway outlining the shared features in the treatment trajectory of the four patients is presented in Fig. 1*.* Potential points of intervention for either education, earlier detection and/or interventions for patients who develop FED post-surgery for upper-abdominal cancer are shown.

## Discussion

These four cases demonstrate new-onset disordered eating behaviours (DEB) after the surgical management of upper abdominal (gastrointestinal, GI) cancer. The current nomenclature for eating disorders does not account for these patients; nor are they normal findings in upper abdominal cancer survivors. The cohort differs from the usual or expected weight loss trajectory that most upper abdominal surgical (oesophagectomy/gastrectomy) patients experience.

One of the most common sequelae of total and partial removal of the stomach (gastrectomy) is weight loss, with a number of previous studies showing that percentage body weight loss after gastrectomy ranges from 10 to 20% of the preoperative body weight [10]. There are several mechanical, neural and chemical factors that contribute to weight loss after surgery [11], including malabsorption as a by-product of reduced transit time, dumping syndrome (secondary to severing of a major nerve i.e., vagotomy), incompletely digested contents causing major absorption concerns (diarrhoea, defective fat absorption etc.). Bacterial overgrowth, a result of altered gastrointestinal anatomy, motility and secretion, is thought to induce an inflammatory response, potentially accounting for chronic diarrhoea. Given these factors, weight loss generally occurs early within the first year and then stabilizes. For example, a large scale prospective cohort study on nutritional intake post partial and total gastrectomy for gastric cancer, found that nutritional intake was recovered at 3 months post-surgery [12]. The ongoing problems with weight loss amongst this cohort is therefore not accounted for by poor oral intake, was in excess of surgical norms, failed to stabilize and no patient was able to restore adequate nutritional intake many months post-surgery. In each case, after multidisciplinary consultation (clinical dietitian, surgeon, and psychiatry) there were concerns of a psychological component to their presentations.

DSM-5 considers feeding and eating disorders (FED) to be “*persistent disturbance of eating, or eating related behaviour, that result in the altered consumption or absorption of food and that significantly impair physical health or psychosocial functioning*” [1]. Whilst there was some variation in the type of DEBs (from nutrition therapy interference, to declining nutrition), all in our cohort had life-threatening malnutrition, and so could the DEBs in these upper abdominal cancer survivors be a subset of FED? If so, this group may be similar to the subset of patients experiencing DEBs post bariatric surgery increasingly recognised by other groups [13].

Prevailing assumptions of the psychosocial aetiologies of FED have been found less applicable amongst the older age-of-onset subgroup. In contrast to those with a typical age-of-onset, there is a lesser predominance of extreme personality traits, or the cardinal features of anorexia, namely body dysmorphia and an aesthetically driven pursuit of thinness [14]. Physical comorbidity, marriage conflicts, separation and divorce have been identified as risk factors for late-onset FED [2]. Our cases certainly reflect these findings.

There is some evidence that earlier intervention for AN may improve outcomes [15]; suggesting that perhaps earlier psychiatric input could have reduced the morbidity and mortality experienced in this case series. A recent paper identified that in gastrectomy patients, continuous BMI (body mass index) reduction 1-year postoperatively is correlated with worse prognosis [16], which may indicate that a step wise intervention may be helpful for a broad group of patients. The hope is to increase awareness and advise earlier nutritional and psychological interventions.

Clinicians (dietitians, surgeons etc.) on the primary team could look for similar patients post-operatively (i.e., those failing to restore weight without a physical health cause) and then individualise care pathways according to the setting. If failure to restore weight at pre-determined points are identified, then a system to flag concerns could be of help (e.g., at interdisciplinary meetings). Similarly, hospital based mental health services need to be available and accessible to assist the primary team members to understand and conceptualise the abnormal behaviours, and be prepared to undertake longitudinal assessments if unclear pathology is present on first review. Finally, the broader team may wish to seek specialist eating disorder advice if needed and available.

## Limitations

In this case series, we chose to focus on the psychosocial factors and have therefore not closely examined the details of each patient’s treatment course, which may have contributed to their clinical presentation. Notably, we did not examine the role of specific surgeries, differences in postoperative commencement of oral intake, pre-existing medical comorbidities, or timing of chemotherapy. Nor did the treating multidisciplinary teams specifically, systematically, considered each of the potential biological drivers of post-operative weight loss for each patient. Though surgical investigation and imaging ruled out major anatomical/functional contributors, there was little, if any mention, of the hormonal and neurological factors which may have been affecting the patients’ presentations.

This case series is limited to a very small number of patients, affecting generalizability. Moreover, the largely qualitative nature of the data limits the reliability and validity of the data, as it is subject to reporter sensitivity and bias.

The retrospective nature of the case studies means that our data pool was limited to the information recorded at the time of each patient’s care, with potentially key pieces of data, (with respect to our hypothesis), not having been thought to be of import at the time.

Similarly, a detailed psychodynamic approach to their case formulations, including addressing any existential themes relating to their initial cancer diagnosis is not presented.

## Conclusion

We propose that a small subset of upper GI cancer patients exist that have (a) severe, excessive, weight loss and (b) psychologically mediated disordered eating behaviours, and that this may lead to very poor clinical outcomes. No similar case series was found in the literature at this time and the clinical significance is uncertain. These findings require further validation and/or similar findings in other settings.

## Data Availability

The datasets used and/or analysed during the current study are available from the corresponding author on reasonable request.

## Abbreviations

AN: Anorexia nervosa
ARFID: Avoidant/restrictive food intake disorder
BMI: Body mass index
CL: Consultation liaison
DEBs: Disordered eating behaviours
DSM-5: Diagnostic and statistical manual of mental disorders, 5th edition
ED: Emergency department
FEDs: Feeding and eating disorders
GI: Gastrointestinal
TPN: Total parenteral nutrition
